# Characterisation of a novel chicken-derived H3N3 avian influenza virus detected in China in 2023: Pathogenicity and immunogenicity

**DOI:** 10.1371/journal.pone.0332213

**Published:** 2025-09-23

**Authors:** Yan Wang, Ya-dong Gao, Cheng-hui Jiang, Yao Xi, Ming-xuan Yang, Wei Zhang, Yang-yang Pan, Qiao-ying Zeng

**Affiliations:** 1 College of Veterinary Medicine, Gansu Agricultural University, Lanzhou, PR China; 2 College of Veterinary Medicine, Northeast Agricultural University, Harbin, PR China; University of South Dakota, UNITED STATES OF AMERICA

## Abstract

The poultry industry faces a constant threat from the mutation and transmission of avian influenza viruses (AIVs). While waterfowl and wild birds are natural hosts of H3N3 AIV, reports of H3N3 infections in chickens are limited. However, in 2023, a decline in egg production among laying hens in the Yancheng Region of Jiangsu Province prompted a study. This research aimed to diagnose the aetiology in laying hens through molecular virological methods and characterise the biological properties of the causative pathogens. An H3N3 AIV subtype strain, A/chicken/China/YC01/2023(H3N3), was isolated from chickens exhibiting lesions. Genome sequencing and analysis revealed a novel genetic makeup: the HA gene originated from an H3N8 AIV, the NA gene from an H10N3 AIV*,* and the internal genes from an H9N2 AIV, all circulating in China. Chickens experimentally infected with the isolate showed signs of Harderian gland haemorrhage, nasal mucus, tracheal circumferential bleeding, and lung bleeding and localised necrosis. Histopathological examination confirmed nasal mucosal and tracheal inflammation, lung capillary congestion, liver cell damage, and sparse splenic lymphocytes. Viral shedding was significantly higher in the oropharyngeal cavity, peaking 2–6 days post-infection, compared to the cloaca. For the first time, the immunogenicity of a novel chicken-derived H3N3 subtype AIV was assessed in specific pathogen-free chickens. An inactivated vaccine, prepared from the isolated strain, resulted in antibody titres peaking at 9.6 log_2_ four weeks after immunization. Furthermore, challenges with either the isolated strain or a duck-origin BZ01/2023(H3N3) strain after immunisation did not cause clinical signs or viral shedding on day 4. In conclusion, the isolate H3N3 AIV can replicate in chickens, leading to organ damage and pathogenicity. Crucially, the inactivated vaccine derived from this isolate is highly immunogenic and provides cross-protection against the duck-derived strain.

## Introduction

Influenza A virus (IAV), a single-stranded negative-sense RNA virus with a segmented genome, belongs to the *Orthomyxoviridae* family. It infects a wide range of hosts, including humans, wild birds, poultry, pigs, dogs, horses, cats, cattle, and seals. When two different IAV strains co-infect the same host cell, genetic reassortment can occur, leading to the emergence of novel influenza virus subtypes or genotypes [1]. These new viruses, with their distinct genomic structures and antigenicities, pose significant threats to both the poultry industry and human health. Historically, Influenza A viruses have triggered global pandemics, with four major ones—1918, 2009 (H1N1) [2,3], 1957 (H2N2) [4], and 1968 (H3N2) [5]—attributed to avian influenza virus (AIV), thereby drawing considerable global attention and research on AIVs.

The H3 subtype of AIV is frequently isolated in patients with low-pathogenic infections, with isolation rates peaking during seasons when H3N2 influenza is widespread among humans [6]. Genetic reassortment potentially enables the H3 subtype of AIV to directly infect humans, raising concerns about the transmission routes of zoonotic diseases caused by these viruses [7]. Waterfowl and wild birds are natural, often asymptomatic hosts for the H3 AIV subtype. The first reported H3N3 isolate, A/duck/ALB/712/1978 (H3N3), was obtained in 1976 [8]. Subsequently, isolates have been found in diverse animal populations, including birds [9,10], seals [11], and pigs [12]. A close relationship has been reported between the HA gene of the H3N3 virus isolated from ducks in 1976 and that of the H3N2 strain from the 1968 human pandemic, suggesting that certain mutations in the H3N3 AIV subtype can circulate across different hosts, potentially facilitating interspecies transmission. Furthermore, genetic evolutionary analysis has shown a relationship between Canadian swine isolates and waterfowl H3N3 viruses. Similarly, A/aquatic bird/South Korea/sw006/2016 (H3N3), isolated from wild birds in Korea, was identified as a product of recombination between wild bird and poultry viruses [13]. These findings confirm the widespread transmission of H3N3 viruses among various host populations. More recently, in April 2022, sporadic human infections with the H3N8 subtype of AIV occurred in China’s Henan and Hunan provinces [14,15]. Significantly, in 2023, a highly pathogenic and transmissible novel H3N3 subtype of AIV was detected in a chicken population experiencing declining egg-laying productivity in eastern China [16,17]. These events illustrate the long-term evolution of H3N3 AIV, indicating interspecies transmission from wild birds and waterfowl to poultry, from birds to mammals, and subsequent spillover to humans. With the expanding host range of H3N3 AIVs and escalating public health threats, continuous virus monitoring and comprehensive research on the biological characteristics and vaccine development for H3N3 AIV are imperative.

In February 2023, a commercial egg chicken farm in Yancheng, Jiangsu Province, China, reported a 12% decrease in egg production and a 3% mortality rate. Post-mortem examination of affected chickens revealed lesions such as tracheal haemorrhage with clear blood clots, congested and necrotic lungs, and follicular haemorrhage, strongly suggesting AIV infection (S1 Fig). This study aimed to isolate and identify pathogens from these chickens and investigate the pathogenicity of the isolated strain in specific pathogen-free (SPF) chickens to determine its virulence and potential adverse effects. Moreover, for the first time, we aimed to assess the immunogenicity of the inactivated isolate in SPF chickens, providing a crucial scientific basis for avian influenza prevention and control, as well as public health.

## Materials and methods

### SPF chicken embryos and chickens

SPF eggs were procured from Boehringer Ingelheim Vetmedica Biotechnology Co., Ltd. (Beijing, China) at embryonic day 0. Upon arrival at the laboratory, eggs were immediately placed in an incubator set at 37.5 - 37.8°C and 60% humidity, with an automatic turning mechanism rotating them every 1–2 h. Some eggs were incubated until embryonic day 10 for pathogen isolation under strict aseptic conditions. The remaining eggs were incubated until hatching. After hatching, the SPF chickens were housed in a controlled-environment isolator. The isolator temperature was maintained at 32–35°C during the first week, then gradually reduced by approximately 3°C per week until it reached a constant 20–22°C for the rest of the study. Relative humidity in the isolator was consistently maintained at 50–60%. Chickens had free access to sterile feed and water and were subsequently utilised for pathogenicity and immunogenicity studies of the isolated strain.

### Virus isolation and identification

For pathogen isolation, fifteen pooled samples—comprising trachea, lungs, and livers—from naturally infected chickens were homogenised in sterile phosphate-buffered saline (PBS) solution (0.02 mol/L, pH 7.4) using a grinding mill (Retsch MM400; Verder Retsch Shanghai Trading Co., Ltd.) at 30 Hz and 4°C for 2 min. The homogenate was then centrifuged at 4°C with a gradient, and the resulting supernatant was collected and filtered through a needle filter (Millex®-GP, Code No. SLGP033R; Millipore, MA, USA).

These processed samples were subsequently used to infect five 10-day-old SPF chicken embryos via the allantoic sac, following published methods [18]. The infected embryos were incubated at 37°C for 3–5 d, and embryonic fluid was collected from both dead and surviving embryos. This process was repeated for three generations (P1 to P3), after which the P3 generation culture was aliquoted and stored at −80°C. A control group, treated identically but inoculated with sterile PBS solution (0.02 mol/L, pH 7.4) was included.

Total RNA was extracted from P3 samples using TRIzol reagent (Invitrogen, Carlsbad, CA, USA) and reverse-transcribed into cDNA using a TIANScript II RT Kit (TIANGEN, Beijing, China). Newcastle disease virus (NDV), AIV, and avian infectious bronchitis virus (IBV) were detected using commercial reverse transcription quantitative PCR (RT-qPCR) kits (Harbin Guosheng Biotechnology Co., Ltd, Harbin, China), following manufacturer’s instructions. Egg drop syndrome virus (EDSV) was detected using the TaqMan fluorescence-based quantitative PCR method reported by Ma et al. [19], using primers and probes synthesised by Takara (Dalian, China).

To determine the HA subtype of the P3 culture, a 1% suspension of chicken red blood cells was prepared according to Appendix 3702 of the *Pharmacopoeia of the People’s Republic of China* 2020. The haemagglutination inhibition (HI) test, as described in Appendix 3404, was performed using H5, H7, and H9 subtype AIV-positive sera (Harbin Guosheng Biotechnology Co., Ltd., Harbin, China) and H3 subtype AIV-positive sera (a gift from the National Avian Influenza Reference Laboratory, Harbin, China).

### Whole genome amplification and sequencing

To amplify the complete viral genome, nucleic acid extracted from the P3-passaged viral fluid served as a template. The universal influenza virus primer unit 12 was used for reverse transcription into cDNA. Subsequently, eight primer pairs, as reported by Hoffman [20], were employed to amplify the respective viral genome fragments. The resulting amplicons were purified using a PCR purification kit (MiniBEST Agarose Gel DNA Extraction Kit Ver.4.0; Code No. 9762; Takara) and individually ligated into the pMD™19-T vector (Takara). Finally, these constructs were sequenced using an ABI PRISM 3700 DNA analyser (Applied Biosystems).

### Sequencing analysis

Representative reference strain sequences, selected based on virus isolation location, time, and host, were downloaded from the National Center for Biotechnology Information (https://www.ncbi.nlm.nih.gov/) and Global Initiative on Sharing All Influenza Data (https://www.gisaid.org/) databases. Nucleotide sequences of the isolated strains were then compared and aligned using ClustalX2. A phylogenetic tree was generated using the maximum likelihood method in MEGA7.0 software, employing the Tamura-Nei model, which accounts for variations in transition/transversion rates and base frequency biases. Phylogenetic relationships were assessed with 1000 bootstrap replicates. Finally, DNAStar software was used to analyse the similarity of the strains.

### Ethical statement

#### Experimental environment.

All animal experiments were conducted within a Biosafety Level 3 (BSL - 3) isolator.

#### Ethical approval.

The animal care and use protocols received approval from the Experimental Animal Ethics Committee of Gansu Agricultural University, Lanzhou, China (GSAU - Eth - vmc - 2024 - 008).

#### Animal care.

Throughout the experiment, all experimental animals received diligent care and maintenance, strictly adhering to the ethical and biosafety guidelines mandated by the aforementioned committee.

#### Euthanasia.

In instances, where animals exhibited severe lethargy, inactivity, anorexia, or neurological symptoms, they were promptly anaesthetised with ketamine to minimise suffering, followed by euthanasia via CO₂ asphyxiation.

#### Researcher training.

All the researchers participating in these animal experiments underwent professional training organised by the Laboratory Animal Ethics Committee of Gansu Agricultural University. 

### Pathogenicity test

#### Determination of median embryo infectious dose (EID_50_).

To determine the accurate infective dose for subsequent pathogenicity tests, the P3 virus stock was serially diluted in 10-fold gradients. Four dilutions (10^−7^, 10^−8^, 10^−9^, and 10^−10^) were prepared. Each dilution was then inoculated into five 10-day-old SPF chicken embryos (100 µL per embryo), and the embryos were incubated at 37°C for 96 h. Following incubation, embryonic fluids were harvested, and the number of HA-positive samples for each dilution was recorded. The EID_50_ of the virus was subsequently calculated using the Reed–Muench method.

#### Pathogenicity of the isolate in SPF chickens.

To examine the pathogenicity of the isolate via mucosal infection, we followed a published methodology [16,21]. We employed randomisation and blinding to ensure the scientific rigour and objectivity of the results. Forty SPF chicken eggs were hatched, and the chickens were reared in isolation until they reached 28 days of age. For randomisation, group assignment was performed using a computer-generated random number table. From the 40 chickens, 30 were randomly selected and divided into experimental and control groups, each consisting of 15 chickens, and maintained under isolated feeding conditions.

A single-blind method was adopted: the researcher monitoring chicken conditions (including disease incidence, mortality, and tissue lesions) was unaware of each chicken’s group assignment. To ensure this, chickens were labelled with codes that did not reveal their group information. Chicks in the experimental group were each intranasally inoculated with 0.2 mL of the viral suspension (viral content of 2 × 10^6.0^ EID_50_), while those in the control group received an identical dosage of sterile PBS via the same route.

The incidence of disease and mortality was observed daily until the end of day 14. To calculate the mean death time, the chickens that died at each observation point were counted towards the mortality at the subsequent observation. Surviving chickens were euthanised on day 14 post-challenge. During the first week after the challenge, chickens were observed at least twice daily, and at least once daily thereafter.

Concurrently, five chickens from each group were randomly selected and euthanised 4 d post-inoculation for tissue lesion observation. Thereafter, the nasal turbinate, trachea, lungs, liver, and spleen were fixed in 10% formaldehyde solution for 7 d, processed into paraffin sections, stained with haematoxylin and eosin, and observed under an optical microscope.

Additionally, throat and cloacal swabs were collected on days 2, 3, 4, 5, 6, 8, and 10 post-inoculation for viral titration to assess shedding status. Blinding was maintained throughout sample collection and analysis. Only after all data were collected and initially processed was the group information unblinded for further statistical analysis.

### Vaccine preparation of the isolated strain

To prepare an oil emulsion-inactivated vaccine, the P3 generation virus stock was purified to the F6 generation using the chicken embryo endpoint dilution method. Its potency was determined and adjusted to 10^8.5^ EID_50_/0.1 mL. For inactivation, formaldehyde (1‰ v/v) was added, and the mixture was incubated at 37°C on a shaker at 200 rpm for 18 h. The inactivated virus was then subjected to three blind passages in chicken embryos. It was deemed suitable for use as the antigen solution only if embryos showed no lesions and the HA titres of the harvested embryo fluids were all negative.

To prepare the aqueous phase of the vaccine, four parts of sterile Tween-80 and 96 parts of the antigen solution were thoroughly mixed in a dispensing tank. This mixture was stirred with a motor for 20–30 min until the Tween-80 was completely dissolved. For emulsification, two parts of white oil adjuvant was placed in an emulsification tank and stirred slowly with a motor. Subsequently, one part of the aqueous phase was slowly added, and the mixture was stirred at 4000 rpm for 30–40 min. Upon examination, the final vaccine appeared as a milky white oil-in-water emulsion. Its good stability and suitability for use were confirmed by the absence of layering after centrifugation at 1610 × *g* for 15 min.

### Immunisation and observation

To assess the immunogenicity of the inactivated isolated strain, we followed the potency test method for inactivated H9 subtype AIV vaccine [22]. Sixty 7-day-old SPF chickens were selected. Of these, 30 were randomly assigned to the immunisation group and subcutaneously inoculated with the inactivated vaccine at a dose of 0.25 mL/feather.

At 28 days old, 10 chickens were randomly selected from the immunisation group and each intranasally inoculated with 0.2 mL/feather of the viral suspension of the isolated strain (2 × 10^6.0^ EID_50_). Another 10 chickens from the same group were inoculated with the A/duck/Shandong/BZ01/2023(H3N3) strain (isolated from the Cherry Valley Duck of Binzhou, Shandong, China, in May 2023 by our laboratory) at the same dose.

Similarly, the challenge control group, comprising 20 randomly selected unvaccinated chickens, was divided into two subgroups of 10 each and intranasally inoculated with the same doses of either the isolated strain or the A/duck/Shandong/BZ01/2023 (H3N3) strain. The blank control group consisted of 10 chickens that received neither vaccine nor virus inoculation.

Blood samples were collected from the immunisation group before immunisation and at 1, 2, 3, and 4-weeks post-immunisation to isolate serum and detect HI antibodies.

Daily clinical observations and mortality assessments were recorded for all experimental chickens post-challenge until the experiment concluded on day 14. Chickens that died during the observation period were counted as mortalities at the subsequent observation time-point for computing the mean time of death. Surviving chickens were humanely euthanised on day 14 post-challenge. Throughout the initial week following the challenge, chickens were monitored at least twice daily, and at least once daily thereafter.

At 4 d post-challenge, samples from the oral and cloacal cavities of each group were collected using cotton swabs to assess shedding status. For each sample inoculated into chicken embryos, if at least one embryo had an HA titre of >1:16 (microtitration method), it was considered positive for virus isolation; for samples negative for virus isolation, a blind passage was performed before determination.

To reduce observer bias, a single-blind method was employed in this study. The personnel responsible for observing and recording the experimental data were unaware of the group assignments of each chicken. Grouping information was only known to the researchers involved in the initial randomisation and the virus/vaccine inoculation procedures.

### Statistical analysis

Basic analysis and chart production of the experimental data were performed using GraphPad Prism software (version 9.5; GraphPad Software, La Jolla, CA, USA). Subsequently, statistical analyses were conducted employing the general linear model within SPSS Statistics software (version 25; IBM Corporation, Armonk, NY, USA). The statistical analysis model is as follows:

Model A


$$\text{Y}=\mu +\alpha_{\text{i}}+\beta_{\text{j}}+\epsilon_{\text{ij}}$$

where Y is the observed value; μ is the overall mean, representing the average level of all observations; αi is the effect of the i-th level of the ‘swab type’ factor (e.g., oropharyngeal swab or cloacal swab); βj is the effect of the j-th level of the ‘days after challenge’ factor (e.g., 2 d, 3 d, etc.); and εij is the random error term, representing the unexplained portion of the model.

Model B


$${\text{Y}}_{\text{ijl}}=\mu +\alpha_{\text{i}}+\beta_{\text{j}}+(\alpha \beta {)}_{\text{ij}}+\varepsilon_{\text{ijl}}$$

where Y is the virus titre; μ is the the population mean; αi is the effect of different sampling methods (oropharyngeal or cloacal swabs); βj is the effect of different treatment groups (immunised and control groups); (αβ)ij is the interaction effect between the i-th level of the sampling method and the j-th level of treatment groups; and εijl is the random error term.

Results are presented as the mean ± standard deviation. A statistically significant difference was defined as **P* *< 0.05, and a highly significant difference as **P* *< 0.01.

## Results

### Isolation and identification of H3 subtype AIV

**Fig 1:**
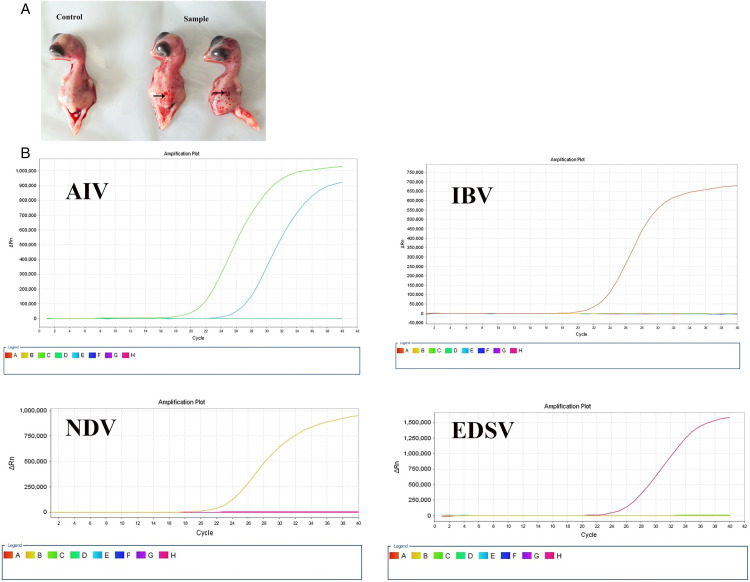
Isolation and identification of avian influenza virus (AIV). (A) Chicken embryo pathology. Diseased chicken samples were inoculated into 10-day-old specific pathogen-free (SPF) embryos via the allantoic cavity. At 72 h post-inoculation, infected embryos (Sample) showed haemorrhage (the black arrow indicates the haemorrhage), while control embryos (Control) were lesion-free. (B) Reverse transcription quantitative PCR (RT–qPCR) detection of multiple avian viruses in P3 cultures of infected embryos. AIV: Sample (C, Ct = 19.52), positive control (E, Ct = 25.29), negative control (D). Newcastle disease virus (NDV): Sample (H), positive control (B, Ct = 20.79), negative control (G). Avian infectious bronchitis virus (IBV): Sample (D), positive control (B, Ct = 20.77), negative control (G). Egg drop syndrome virus (EDSV): Sample (B), positive control (H, Ct = 23.49), negative control (C).

The supernatant from the 15 pooled naturally infected chicken samples was inoculated into chicken embryos. Embryo deaths occurred between 48–84 h post inoculation, with affected embryos exhibiting haemorrhage. In contrast, the control group showed no embryo mortality or apparent lesions (Fig 1A). RT-qPCR analysis of the P3 chicken embryo culture indicated specific AIV nucleic acid positivity (Ct = 19.52 ≤ 35), while simultaneously confirming negativity for NDV (No Ct), IBV (No Ct), and EDSV (No Ct). This definitely confirmed AIV infection (Fig 1B).

Furthermore, HI test results showed that the HI titre of the P3 generation culture with H5, H7, and H9 subtype AIV-positive sera was < 3 log_2_, indicating a negative result. Conversely, the culture showed a titre of 10 log_2_ with H3 subtype AIV-positive serum, preliminarily identifying the isolated strain as an H3 subtype AIV (Table 1).

**Table 1:** HI test results.

Positive serum for AIV	HI antibody titre (log_2_)	
	Experimental group	Control group
H5 subtype	0	0
H7 subtype	0	0
H9 subtype	0	0
H3 subtype	10	0

AIV, avian influenza virus.

### Analysis of the whole genome sequence of the virus

The complete gene sequences of all eight segments of the isolate were obtained through gene cloning, sequencing, and assembly. These sequences have been uploaded to GenBank with accession numbers: PP977193, PP977197, and PP998321–PP998326.

BLAST alignment of the nucleotide sequences of these segments revealed distinct origins for different genes. Specifically, the HA and M genes of the isolate exhibited the highest similarity to the H3N8 virus circulating in chicken populations in Jiangsu Province in 2022. In contrast, the NA and NP genes showed the highest similarity to the H10N3 virus isolated from chicken populations in China in 2021. Other genes displayed the highest similarity with the H9N2 AIV subtype (Table 2).

**Table 2:** Genome BLAST analysis of YC01/2023(H3N3) avian influenza virus.

Gene	Highest similarity strain	Accession No.	Similarity (%)
HA	A/chicken/Jiangsu/B314/2022(H3N8)	EPI2047625	98.99
M	A/chicken/Jiangsu/B314/2022(A/H3N8)	EPI2047636	99.50
NA	A/chicken/Jiangsu/HY401/2021(H10N3)	EPI1884776	99.00
NP	A/chicken/China/146-5/2021(H10N3)	OL636801.1	100.00
PB2	A/Environment/Taizhou/03/2019(H9N2)	EPI1607454	98.03
PB1	A/Environment/Jiangsu/zj1055/2021(H9N2)	EPI1884540	98.91
PA	A/Environment/Jiangsu/zj1055/2021(H9N2)	EPI1884541	99.28
NS	A/chicken/Fujian/7.20-FZHX0013-O/2017(H9N2)	MW102180.1	98.45

Therefore, these findings confirm the isolation of a novel H3N3 AIV strain, which has been named A/chicken/China/YC01/2023(H3N3) [abbreviated as YC01/2023(H3N3)].

### Phylogenetic analysis of HA and NA genes and the viral gene reassortment hypothesis

**Fig 2:**
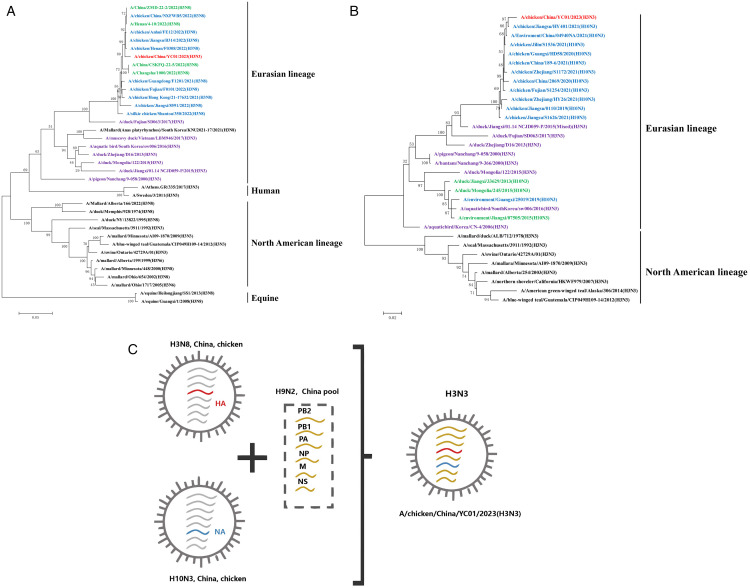
Phylogenetic analysis of the HA and NA genes, and reassortment hypothesis. (A) HA gene phylogenetic analysis: YC01/2023(H3N3) isolate (red); 2021–2022 Chinese avian H3N8 isolates (blue); 2022 Chinese human-origin isolates (green); other H3N3 reference strains in the same clade (purple); and other clade strains (black). (B) NA gene phylogenetic analysis: YC01/2023(H3N3) isolate (red); 2019–2021 Chinese avian H10N3 isolates (blue); other H3N3 reference strains in the same clade (purple); pre-2019 H10N3 reference strains (green); and other clade strains (black). (C) Recombination pathway and segment source hypothesis. The novel H3N8 virus prevalent in Chinese chickens reassorted with H10N3 and H9N2 viruses, leading to the generation of the China/YC01/2023(H3N3) strain.

Phylogenetic analysis revealed that the HA gene of the isolated strain belonged to the Euro-Asian avian evolutionary branch, aligning with the H3N8 virus prevalent in China from 2021 to 2022 [16,17,23,24]. This gene was genetically closest to the chicken-derived strain A/chicken/Jiangsu/B314/2022(H3N8), also isolated from Henan and Anhui, showing a genetic similarity of 99.0%. Genetic similarity with other H3N8 viruses within the same branch ranged from 96.9–99.0%, whereas similarity with the H3N3 reference strain ranged from 85.2–90.7% (S2 Table and S2 Fig). The higher genetic similarity with reference strains from similar years indicates a certain temporality in evolution. In contrast, within the North American lineage, human- and horse-derived strains formed an independent branch, exhibiting a distant genetic relationship with the isolated strains (Fig 2A).

Similarly, the NA gene of the isolated strain also belonged to the Euro-Asian avian evolutionary branch. It was genetically closest to the H10N3 virus prevalent in China from 2019 to 2021, specifically to the chicken-derived strain A/chicken/Jiangsu/HY401/2021(H10N3), with a genetic similarity of 99.0%. Genetic similarity with other H10N3 viruses in the same branch ranged from 97.6–99.0%, whereas similarity with the H3N3 reference strain ranged from 82.5–99.5% (S3 Table and S3 Fig; Fig 2B).

Regarding the internal genes, the isolated strain showed the highest genetic similarity with the M gene of A/chicken/Jiangsu/B314/2022(A/H3N8) and the NP gene of A/chicken/China/146-5/2021(H10N3). Both of these parent strains contained internal genes derived from H9N2 AIV. Furthermore, the other internal genes of the isolated strain displayed the highest similarity with those of H9N2 AIV in China. This comprehensive phylogenetic analysis strongly suggests that the isolated China/YC01/2023(H3N3) strain emerged from genetic recombination involving the novel H3N8 virus prevalent in Chinese chicken populations, and H10N3 and H9N2 viruses also circulating in China (Fig 2C).

### Clinical signs, pathological changes and viral shedding in the experimental group

The viral titre of the P3 virus stock was calculated as 10^8.83^ EID_50_/0.1 mL using the Reed–Muench method (S4 Table). Subsequently, chickens in the experimental group were each intranasally inoculated with 0.2 mL of the viral suspension (2 × 10^6.0^ EID_50_).

Two days post-inoculation, 8 of the 10 chickens in the experimental group exhibited clinical signs, including ruffled feathers, neck retraction, white watery faeces, and swollen conjunctiva. No fatalities were observed throughout the experiment, which concluded on day 14. All 10 chickens were humanely euthanised on day 14 post-challenge.

**Fig 3:**
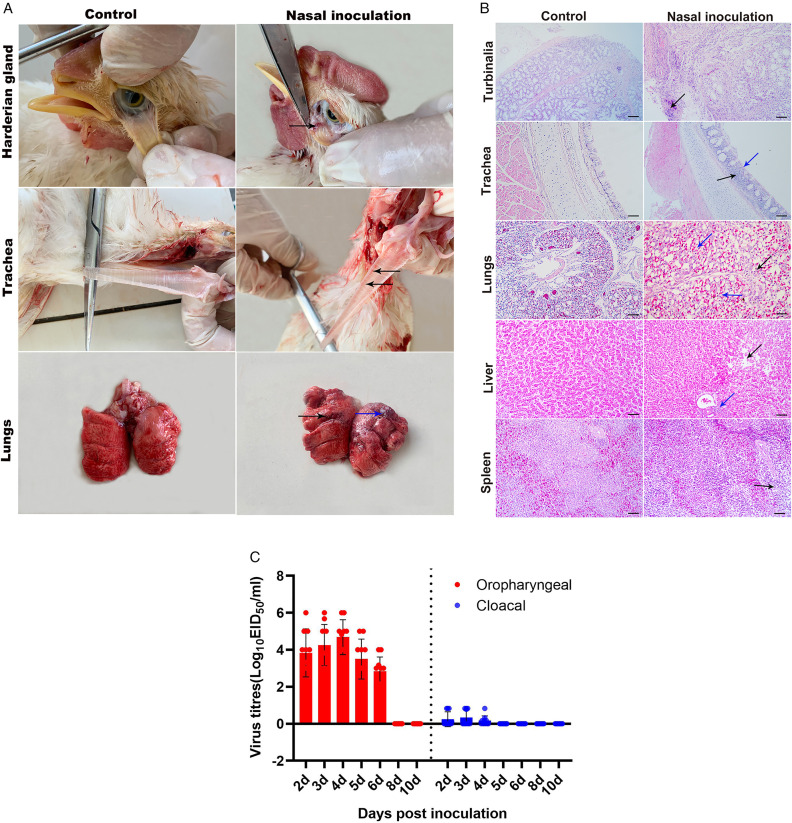
Organ lesions, histopathological changes, and viral shedding in experimentally infected chickens. (A) Macroscopic pathology of the Harderian gland, trachea, and lungs. In the intranasal inoculation group (top to bottom): black arrows indicate unilateral Harderian gland haemorrhage, slight circumferential tracheal bleeding, and mucus-accompanied pulmonary bleeding; the blue arrow indicates local pulmonary necrosis. No lesions were observed in the control group. (B) Histopathological images of nasal turbinate, trachea, lung, liver, and spleen tissues (H&E staining, 200 × ; scale bar = 50 μm). Intranasal inoculation group – Nasal cavity: Obvious inflammatory cell infiltration (black arrow). Trachea: Partial ciliary loss in mucosal epithelial cells (blue arrow), extensive inflammatory cell infiltration in the mucosa (black arrow). Lungs: Capillary congestion and bleeding (blue arrow), slight inflammatory cell infiltration (black arrow). Liver: Hepatocyte vacuolar degeneration, mild inflammatory cell infiltration around the central vein (blue arrow), focal necrosis in some areas (black arrow). Spleen: Sparse lymphocyte distribution in the white pulp (black arrow). No obvious pathological changes were observed in the control group. (C) Viral shedding in chickens after inoculation with YC01/2023(H3N3) AIV (avian influenza virus). Post-inoculation, oropharyngeal viral titres from day 2 to day 10 are indicated by red bars, and cloacal titres by blue bars.

Necropsy performed 4 d post-inoculation revealed that some chickens had unilateral Harderian gland haemorrhage, mucus in the nasal cavity, slight circumferential bleeding in the trachea, slight bleeding with mucus and localised necrosis in the lungs (Fig 3A).

Histopathological observations further revealed significant inflammatory cell infiltration in the nasal cavity, partial loss of cilia in the tracheal mucosal epithelial cells with abundant inflammatory cell infiltration in the mucosal layer, lung capillary congestion with epithelial cell swelling and partial inflammatory cell infiltration, prominent hepatocyte swelling with vacuolar degeneration, infiltration of a few inflammatory cells around the central vein with some cells undergoing necrosis, and sparse lymphocytes in the splenic white pulp (Fig 3B). In contrast, the control group exhibited no characteristic clinical signs or significant pathological changes in the nasal cavity, trachea, lungs, liver, or spleen.

Analysis using Statistical Model A revealed highly significant differences in viral shedding among different treatments. Specifically, viral shedding in the oropharynx was significantly higher than that in the cloaca (**P* *< 0.01), with peak shedding occurring between days 2 and 6 post-inoculation (Fig 3C).

### Evaluation of inactivated vaccine immunogenicity

#### HI antibody detection.

**Fig 4:**
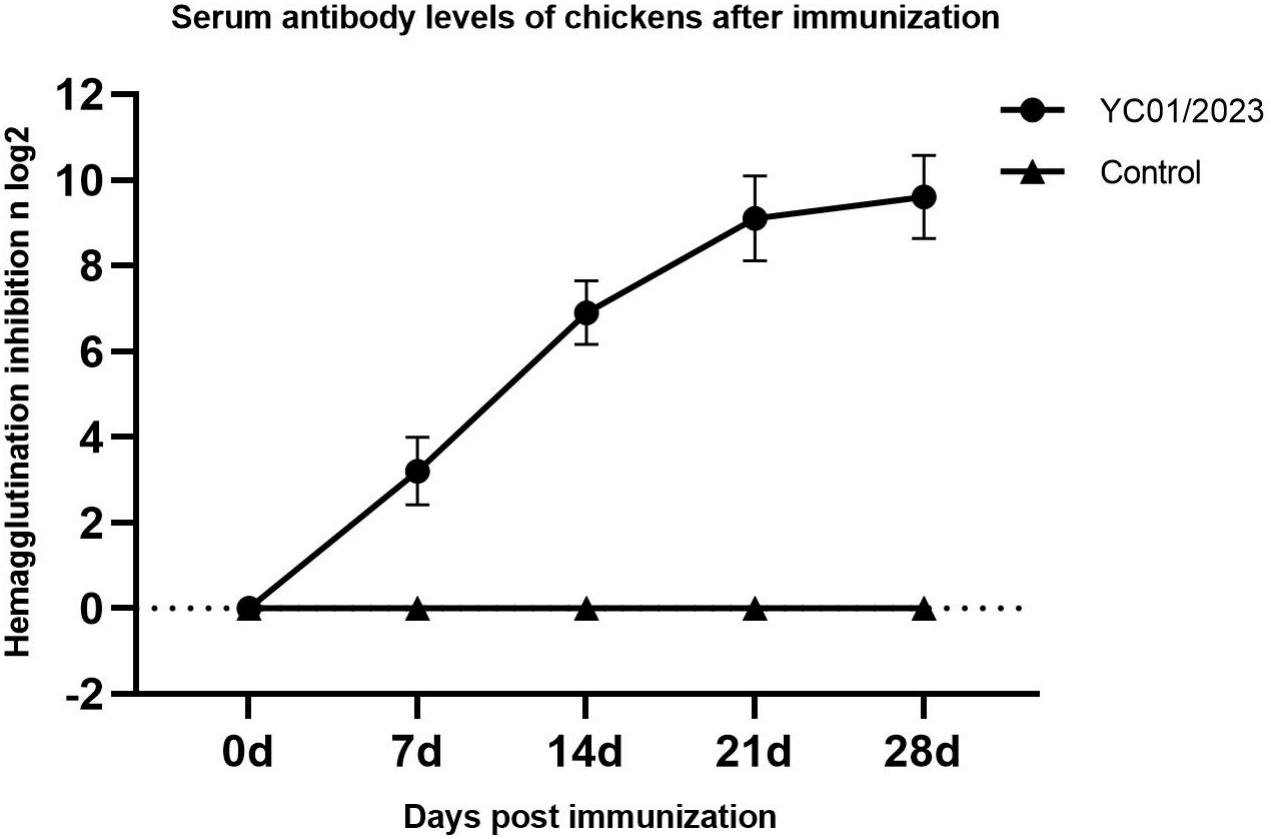
Serum antibody levels in specific pathogen-free (SPF) chickens after immunisation. Black circles denote the mean haemagglutination inhibition (HI) antibody titres of chickens immunised with inactivated vaccines from immunised isolates at 0 (pre-immunisation), 7, 14, 21, and 28 d post-immunisation. Black triangles represent the mean HI antibody titres of the non-immunised control group at corresponding time points.

Following immunisation, the immunised group exhibited an increasing trend in antibody levels, with a peak titre of 9.6 ± 0.97 log_2_ observed after 4 weeks. Statistical analysis using Model A indicated that the antibody titres in the immunised group were significantly higher than those in the control group (**P* *< 0.01) (Fig 4).

#### Clinical observation and viral shedding monitoring.

Following challenge, no clinical signs of disease were detected in the immunised or negative control groups until the experiment’s conclusion. Similar to observations from the pathogenicity studies of the isolated strains, chickens in both the YC01/2023(H3N3) challenge and BZ01/2023(H3N3) challenge control groups exhibited symptoms. Specifically, 8 of 10 chickens in the YC01/2023(H3N3) challenge control group and 9 of 10 chickens in the BZ01/2023(H3N3) challenge control group showed ruffled feathers, neck retraction, white watery faeces, and swollen conjunctiva. No fatalities were observed in any experimental group throughout the experiment. All surviving chickens were humanely euthanised on day 14 post-challenge.

**Fig 5:**
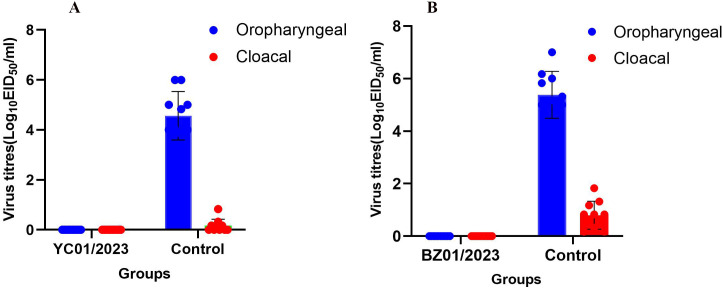
Viral shedding following challenge inoculation post-vaccination. Viral shedding 4 d after inoculation with the (A) YC01/2023(H3N3) and (B) BZ01/2023(H3N3) strains. Oropharyngeal viral titres are represented by blue bars, and cloacal titres by red bars.

On day 4 post-inoculation with the YC01/2023 (H3N3) strain, virus shedding was detected in all animals in the control group, whereas no virus shedding was detected in the immunised group (Fig 5A). A comparable pattern of virus shedding was observed on day 4 post-inoculation with the BZ01/2023 (H3N3) strain (Fig 5B).

Statistical analysis utilising Model B demonstrated that the virus titre in the control group was highly significantly greater than that in the immunised group (P < 0.01). Furthermore, the virus titre in oral swabs from the control group was highly significantly greater than that in cloacal swabs (P < 0.01).

## Discussion

Waterfowl and wild birds are natural hosts of H3N3 AIV [25,26]. In 2023, sporadic reports of chickens carrying H3N3 AIV emerged in China [16,17]. In this study, we successfully isolated and identified a novel strain of H3N3 AIV, designated YC01/2023(H3N3), from a chicken flock experiencing decreased egg production in the Yancheng Region of China during 2023. Genetic analysis revealed that the HA gene of YC01/2023(H3N3) originated from an H3N8 virus prevalent in China in 2022, while its NA gene is closely related to an H10N3 virus isolated from chickens in China in 2021. Furthermore, its internal genes are derived from H9N2 AIV in China, confirming it is a novel recombinant AIV. Within the haemagglutinin (HA) protein of YC01/2023(H3N3), amino acids at positions 226 and 228 remained glutamine (Q) and glycine (G) respectively, lacking the characteristic mutations (Q226L, G228S) associated with mammalian adaptation. Based on these well-established markers, the immediate zoonotic risk of the YC01/2023(H3N3) strain might be comparatively low.

The pathogenicity and epidemiological role of H3 AIV subtypes carry significant public health implications. Historically, their role in epidemiology has often been overlooked due to their relatively low pathogenicity in clinical settings. However, analysis of the genetic evolution of H3 subtype AIVs in specific regions of China from 2011 to 2015 by He et al. [27] speculated that these viruses have undergone complex gene exchange with H5 and H7 subtype AIVs. Mao et al. further indicated that novel H3N3 AIVs exhibit higher pathogenicity compared to H3 viruses found in wild birds [17,28]. In our study, infection of SPF chickens with the YC01/2023(H3N3) strain resulted in unilateral Harderian gland haemorrhage, severe pathological damage to tissues like the trachea, lungs, and liver, and disruption of respiratory mucosa integrity. Previous studies highlight that such respiratory mucosa disruption can elevate the risk of secondary infections in poultry. We, therefore, hypothesise that this damage may render chickens more susceptible to secondary infections by other pathogenic microorganisms. These secondary infections have the potential to lead to more severe clinical manifestations, including respiratory diseases, decreased egg production, and egg yolk peritonitis, which could explain the higher mortality observed in naturally infected chicken flocks compared to those infected artificially. Future research should include testing for the H3 virus in ovaries and screening for other potential secondary pathogens to clarify the causes of these clinical signs. Sun et al. [16] demonstrated that the viral titre of H3N3 in nasal turbinates and trachea was significantly higher than in the lungs (P < 0.05). They also detected the virus in organs such as the Harderian gland, thymus, and brain. These findings align with the pathological changes observed in our study, indicating extensive tissue tropism of the virus in chickens, leading to systemic infection. As observed by Sun et al. [29], chickens experimentally infected with a novel H3N8 AIV exhibited swollen and hyperaemic eyelids, mild respiratory symptoms, and tissue damage, including necrosis in the Harderian gland, nasal turbinates, and trachea, as well as bronchopneumonia. Efficient viral replication was detected in these tissues, similar to our YC01/2023(H3N3) isolate. These parallel findings highlight the necessity for laboratory detection methods to differentiate between strains and underscore the importance of comparative studies on the virulence of H3N3 and H3N8 AIV. Additionally, our isolated strain originated from 263-day-old Hy-Line Brown layers, while other breeds in the area did not show symptoms or had low incidence rates. This suggests varying susceptibilities among different chicken breeds to the virus, necessitating further experimental studies.

Sun et al. [16] showed that novel H3N3 AIV could be transmitted aerially within chicken flocks, potentially becoming a new dominant epidemic virus in poultry. Large-scale vaccination is considered one of the fastest and most effective measures to control avian influenza, and China has effectively reduced the prevalence of AIVs, including H5, H7, and H9, through vaccination [30–32]. Currently, no commercial vaccines are available against H3N3 AIV. Reduction in shedding and disease prevention are key indicators of vaccine efficacy [33]. In this study, for the first time, we successfully prepared an oil emulsion-inactivated vaccine using a novel H3N3 AIV. When administered to SPF chickens, this vaccine elicited high-level antibodies by day 14, peaking by day 28 with an average titre of 9.6 log_2_, demonstrating a robust immune response. Furthermore, the immunised group of chickens did not exhibit virus shedding following challenge with the YC01/2023(H3N3) strain and remained healthy throughout the trial. This indicates that the vaccine provides good immunoprotection, capable of inhibiting the replication of the isolated strain in the host and reducing virus spread.

Notably, using SPF chickens in this study presents certain limitations. SPF chickens are genetically homogeneous and raised in a pathogen-free environment, which may not fully represent the immune response and disease resistance of commercial chicken breeds. Commercial breeds likely exhibit different genetic backgrounds and immune statuses due to factors such as breeding selection and exposure to different pathogens in real-world environments.

Waterfowl, such as ducks, act as a bridge for transmitting the virus to humans or susceptible chickens [34]; AIVs transmitted from waterfowl to chickens exhibit high pathogenicity [35]. Therefore, monitoring and controlling H3 AIV subtypes in duck sources is essential. Following inoculation with the isolate vaccine in this study, the chickens were challenged with the duck isolate bz01/2023(H3N3). No viral shedding was observed in the immunised group on day 4, indicating that chickens immunised with the isolate strain vaccine can also effectively resist the virulent duck strain, thereby reducing wild virus transmission and disease incidence. This study provides important reference data for preventing the spread of novel H3N3 AIV. However, only one duck-derived H3N3 AIV strain (BZ01/2023) was tested. Future research should encompass multiple strains from diverse sources to confirm their cross-protection capabilities and specific immune responses. Additionally, the follow-up duration in this study was relatively short. Future studies should include longer follow-up periods to monitor antibody levels and immune protection effects of the vaccine. These studies will provide a more comprehensive scientific basis for the development and application of vaccines against the novel H3N3 AIV.

In conclusion, we successfully isolated a novel H3N3 AIV strain, YC01/2023(H3N3), which resulted from reassortment between H3N8 and H10N3 AIVs circulating in Chinese poultry. Chickens infected artificially exhibited typical clinical signs and pathological changes. The isolated strain demonstrated good immunogenicity and offered cross-protection against the duck-derived strain, positioning it as a potential candidate for vaccine development. Importantly, this study contributes valuable epidemiological data on novel H3N3 AIV and provides critical insights for the development of vaccines against novel H3N3 AIV.

## Supporting information

S1 Fig Post-mortem lesions of chickens with decreased egg production in Yancheng in 2023. (**A**) The diseased chickens exhibited tracheal bleeding and obvious blood clots; the black arrow indicates a blood clot. (**B**) The blue arrow indicates congested and necrotic lungs, while the black arrow points to follicular haemorrhage.
(TIF)

S2 Table List of reference sequences for nucleotide similarity analysis of the HA gene. (TIF)

S2 Fig Nucleotide similarity analysis of the HA gene. Red highlighting indicates a similarity range of 85.2% to 90.7% with the H3N3 reference strain.
(TIF)

S3 Table List of reference sequences for nucleotide similarity analysis of the NA gene. (TIF)

S3 Fig Nucleotide similarity analysis of the NA gene. Red highlighting indicates a similarity range of 82.5–99.5% with the H3N3 reference strain.
(TIF)

S4 Table Experimental data for determining the median embryo infectious dose (EID_50_). (TIF)
