# Supplementary figures and images for: Characterisation of a novel chicken-derived H3N3 avian influenza virus detected in China in 2023: Pathogenicity and immunogenicity
Source: PLoS One. 2025 Sep 23;20(9):e0332213. doi: 10.1371/journal.pone.0332213 (PMC12456792; doi:10.1371/journal.pone.0332213)

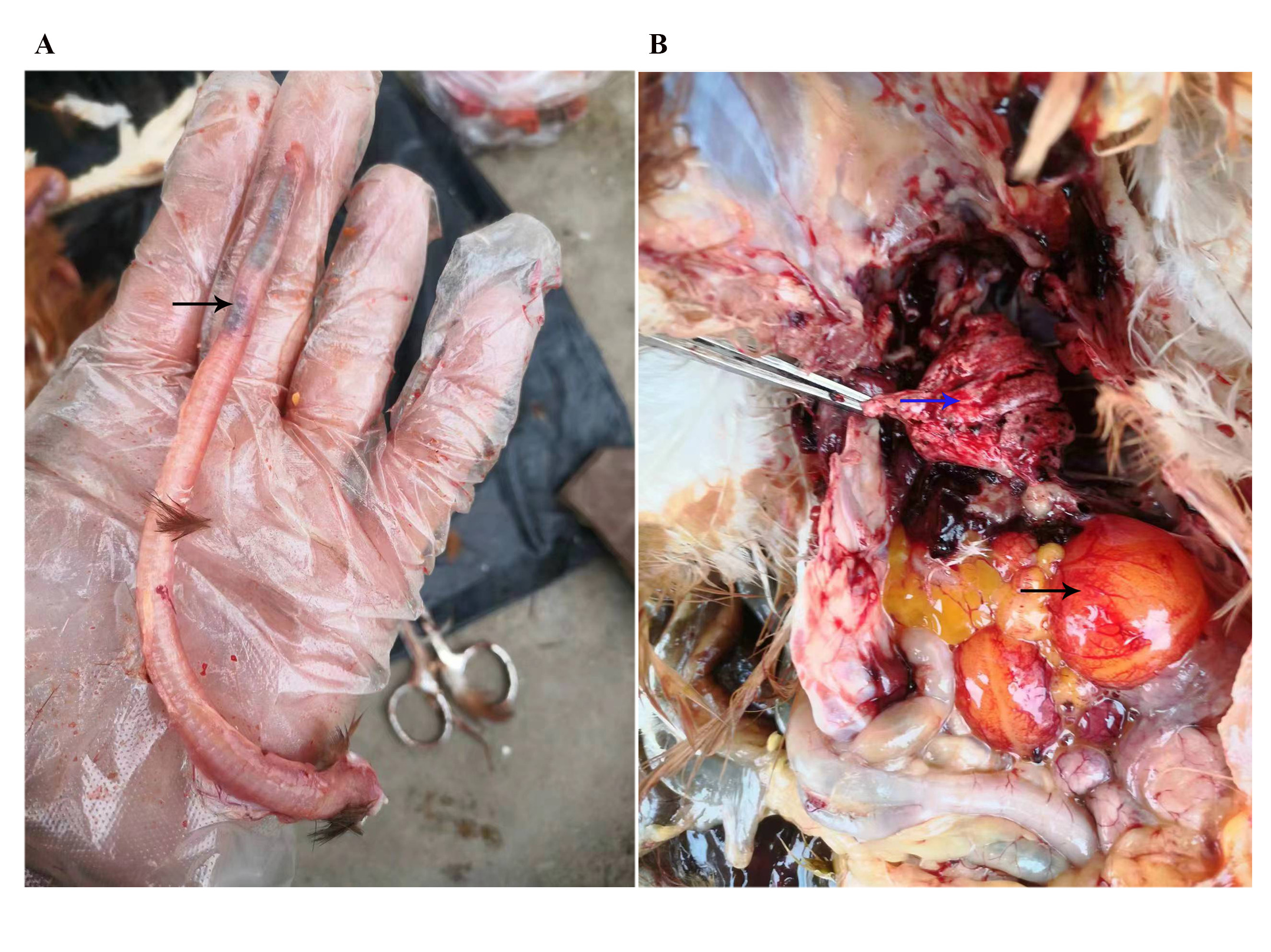

Supplement: S1 Fig — (A) The diseased chickens exhibited tracheal bleeding and obvious blood clots; the black arrow indicates a blood clot. (B) The blue arrow indicates congested and necrotic lungs, while the black arrow points to follicular haemorrhage. (TIF) [file pone.0332213.s001.tif]

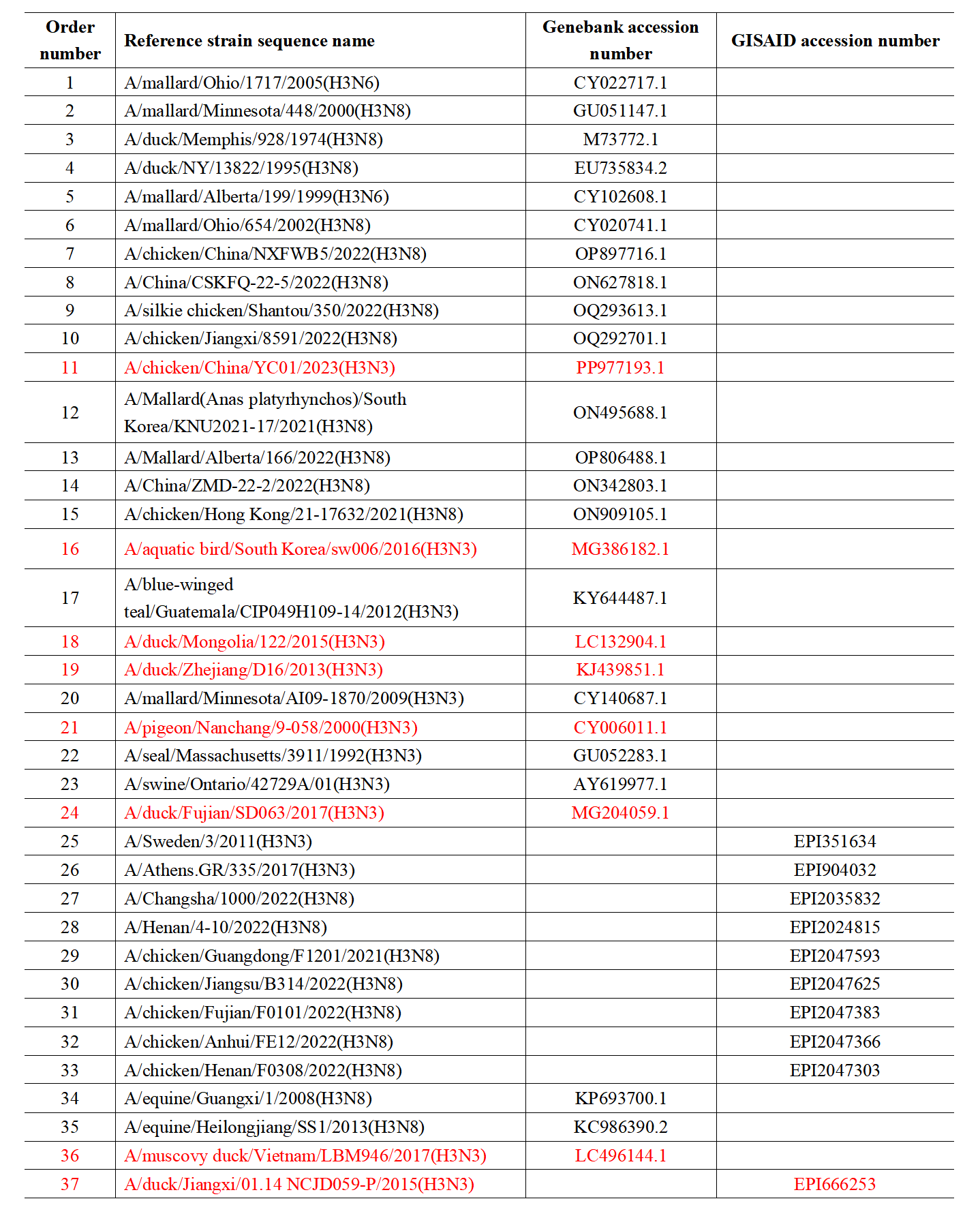

Supplement: S2 Table — (TIF) [file pone.0332213.s002.tif]

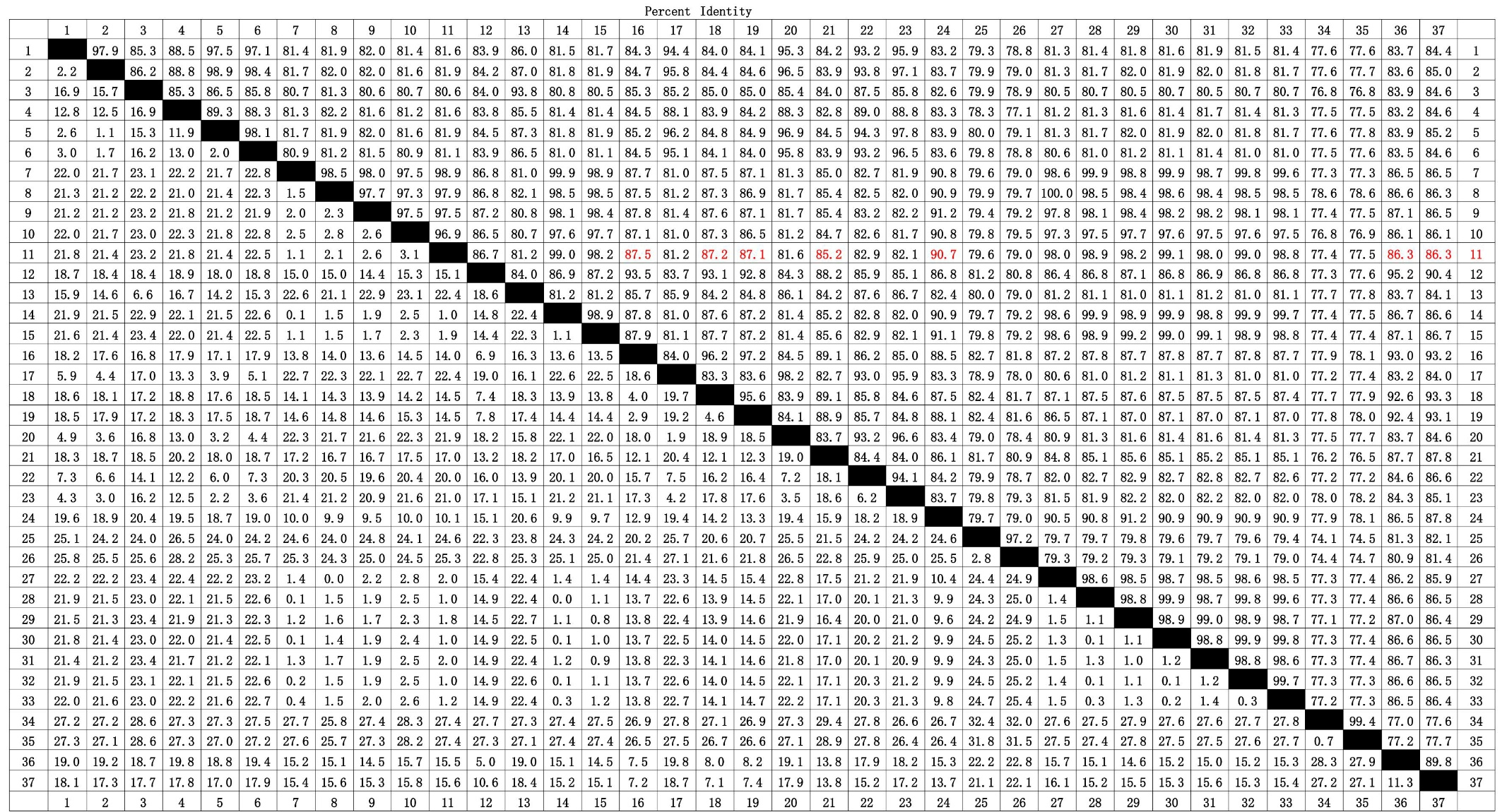

Supplement: S2 Fig — Red highlighting indicates a similarity range of 85.2% to 90.7% with the H3N3 reference strain. (TIF) [file pone.0332213.s003.tif]

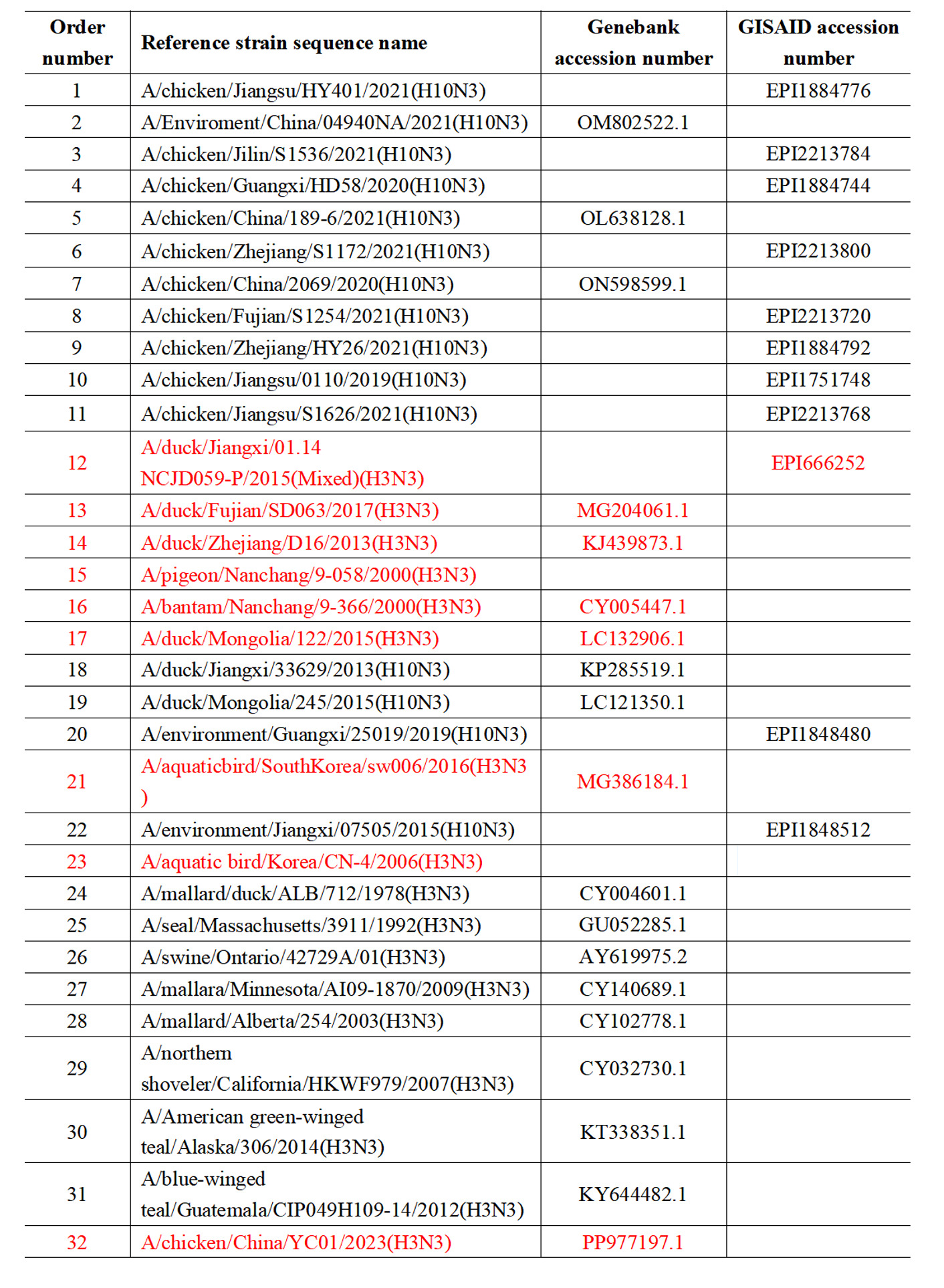

Supplement: S3 Table — (TIF) [file pone.0332213.s004.tif]

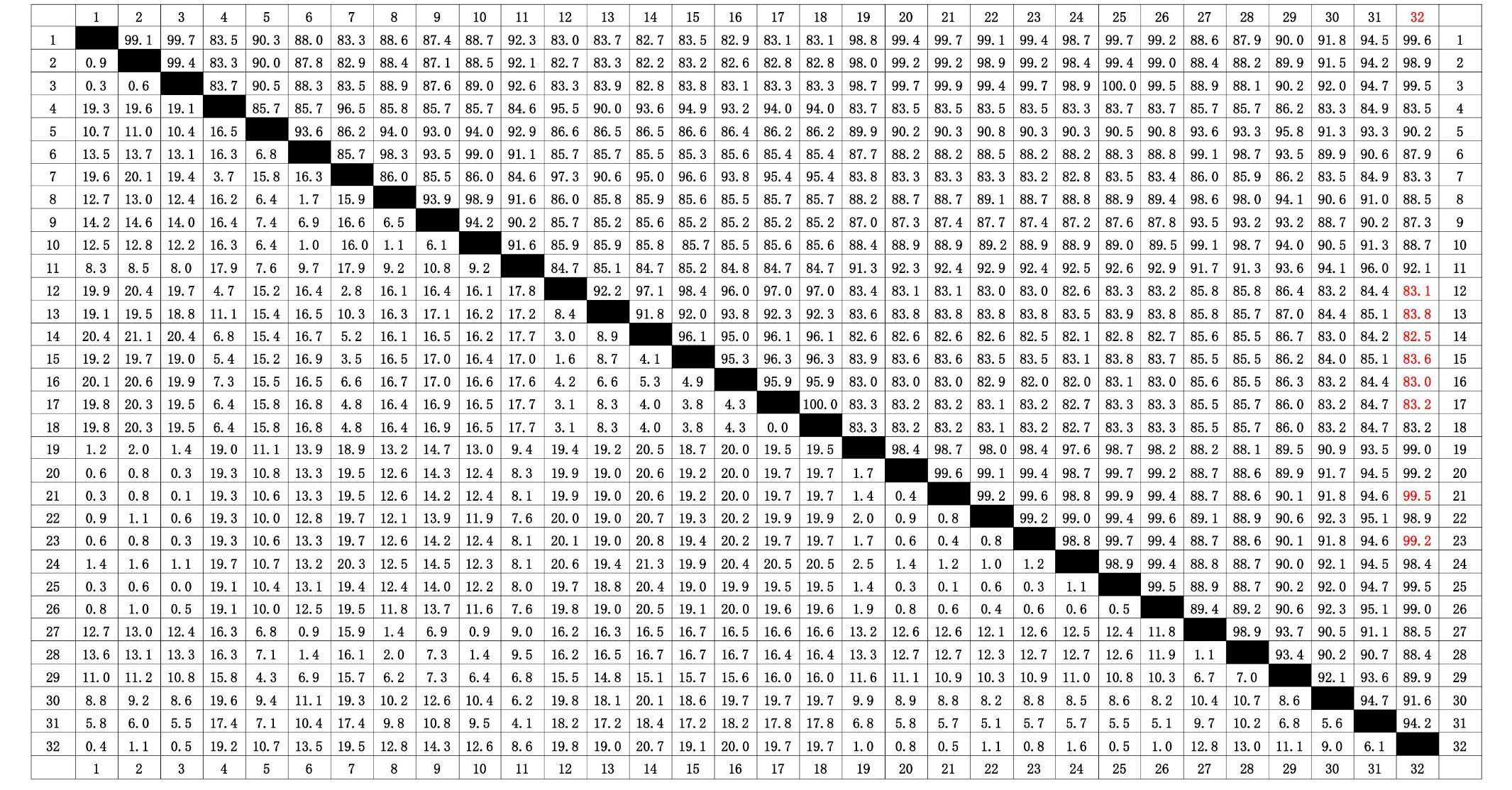

Supplement: S3 Fig — Red highlighting indicates a similarity range of 82.5–99.5% with the H3N3 reference strain. (TIF) [file pone.0332213.s005.tif]

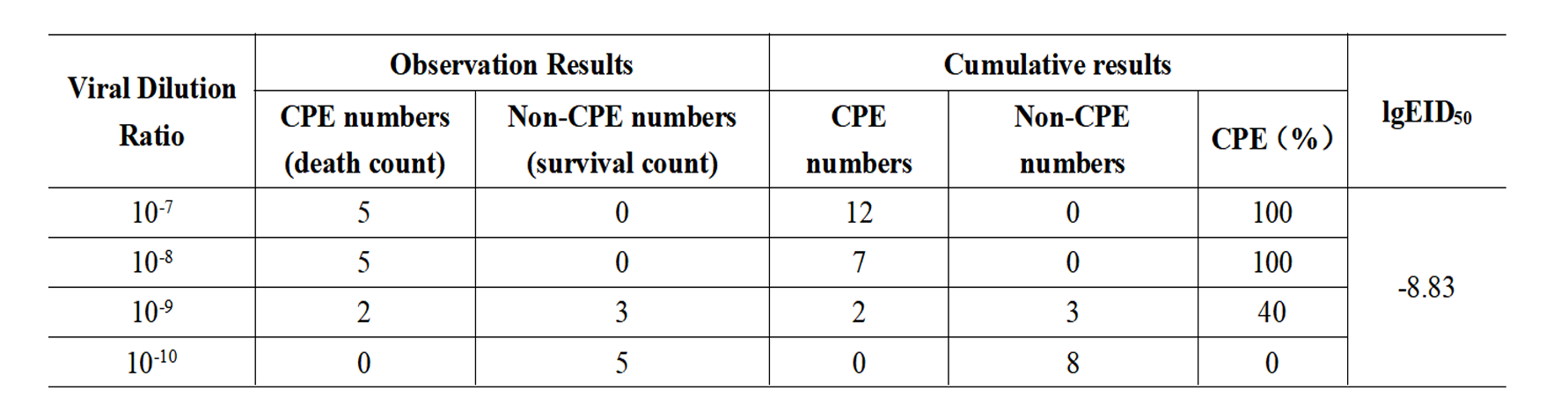

Supplement: S4 Table — (TIF) [file pone.0332213.s006.tif]
